# Rethinking Microbiome of the Built Environment (MoBE) Management: From Pathogen-Centric Control to Microbiome-Informed Engineering

**DOI:** 10.1021/acs.est.6c02788

**Published:** 2026-04-23

**Authors:** Claudia K. Gunsch, Joe Brown

**Affiliations:** † Department of Civil & Environmental Engineering, 3065 Duke University, Durham, North Carolina 27708, United States; ‡ Department of Environmental Sciences and Engineering, 41474 Gillings School of Global Public Health, University of North Carolina, Chapel Hill, North Carolina 27599, United States

**Keywords:** public health, microbiome, built environment

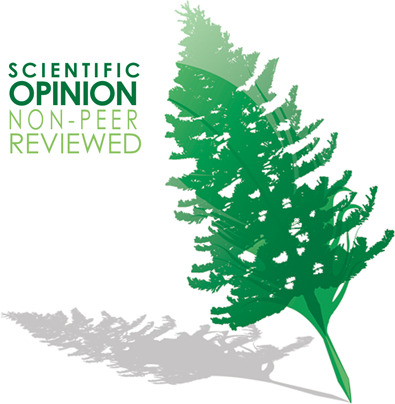

## Graphical abstract

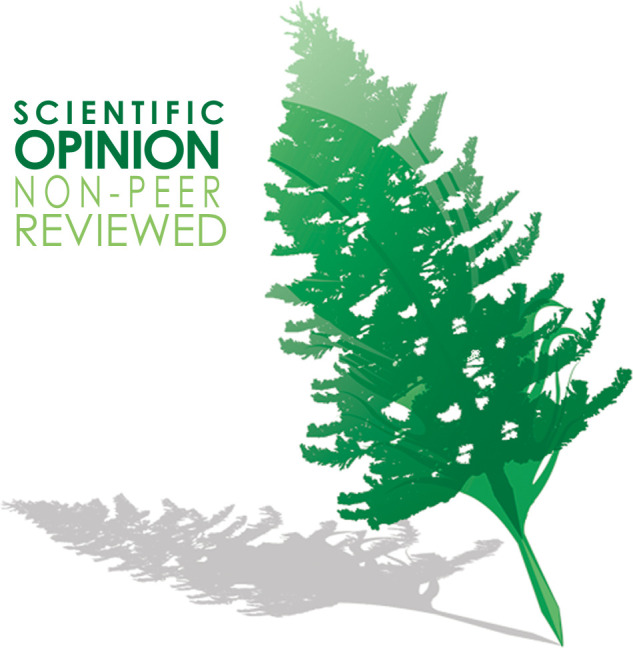

Environmental engineering has historically defined microbial performance through pathogen suppression. Drinking water treatment standards rely on defined log reduction targets and concentration–time relationships to achieve acceptable levels of infection risk.^1^ Ventilation standards and surface disinfection practices similarly prioritize removal or inactivation of specific microbes in isolation.^2^ This pathogen-centric framework has delivered extraordinary public health gains and remains essential in high-risk systems where a relatively small number of well-characterized pathogens present known risks.

However, embedded within this framework is a simplifying assumption, that microbial communities can be treated as isolated pathogen hazards to eliminate, rather than as complex and dynamic ecological systems whose structure and function directly influence pathogen persistence, suppression, and regrowth. Microbial control measures in engineered systems typically do not create sustained sterile environments. Microbial communities change and respond to interventions in a variety of complex and context-dependent ways. The responses of the microbiome to engineering controls can directly affect pathogen control outcomes. For example, interventions may counteract pathogen reduction goals through evolved resistance and rapid recolonization or conversely enhance pathogen suppression through competitive exclusion, niche occupation, or community-mediated inhibition.

## Embracing Complexity

Studies of plumbing systems and indoor environments provide a useful illustration of the microbiome of the built environment (MoBE) and the potential benefits of accounting for it in engineering controls. Water distribution systems, premise plumbing, HVAC, and surfaces host structured microbial communities shaped by materials, hydrodynamics, disinfectant regimes, and occupants,^3–6^ assembling through ecological processes such as environmental filtering, dispersal, competition, and disturbance-driven succession.^7^ In distribution systems, water age, pipe materials, and disinfectant strategies influence microbial diversity and resistance gene abundance,^8–11^ with community restructuring occurring even when pathogen compliance targets are met. Sublethal disinfectant exposure and repeated disturbances may enrich resistance or select stress-tolerant taxa,^12,13^ underscoring the limits of pathogen-centric control.

The next evolution of MoBE management should broaden, rather than replace, pathogen control by incorporating microbiome complexity. Engineering interventions shape not only target organisms but also community structure, function, and selective pressures. Environmental antibiotic resistance frameworks recognize that engineered systems can act as selective environments under certain regimes,^12,13^ where pathogen suppression may coincide with enrichment of resistance reservoirs under chronic sublethal stress. Accordingly, microbial performance should account for both inactivation efficacy and resistance propagation potential.

Ecological stability adds a critical dimension to MoBE management. Infrastructure systems experience repeated disturbances, including hydraulic transients, disinfectant fluctuations, and temperature variation. Stable communities may exhibit predictable recovery, functional redundancy, and resistance to pathogen invasion, whereas unstable communities may undergo turnover that creates niches for opportunistic pathogens and increases uncertainty in exposure outcomes. While stability is not synonymous with safety, neither is disturbance with improved control. Defining baseline variability and disturbance response across systems is therefore essential before stability can be operationalized as a performance metric.

## Challenges and Opportunities

A more inclusive MoBE management paradigm offers both challenges and opportunities, requiring investment in innovation. High-throughput sequencing and functional gene profiling now enable infrastructure-scale microbial characterization.^8,14^ The bottleneck is no longer measurement but interpretation, linking community structure and function to system performance. Predictive frameworks connecting intervention to microbiome shift to pathogen dynamics to exposure consequence and health risk remain limited. There are no validated thresholds for ecological stability, diversity, or resistance potential to complement pathogen log reduction benchmarks, nor a consensus definition of a healthy MoBE, which likely varies across environments. Without such benchmarks, microbiome-informed management cannot be translated into quantitative, risk-based decision frameworks comparable to existing regulatory paradigms.

Current frameworks are optimized for acute hazard mitigation,^1,15^ defining acceptable risk using pathogen end points and exposure assumptions derived from quantitative microbial risk assessment. While effective for preventing outbreaks and reducing waterborne disease, they are not structured to evaluate how ecological stability, disturbance recovery, or resistance selection influence long-term pathogen control and health risks. National assessments emphasize that MoBEs represent a systems-level frontier requiring integration of microbiology, engineering, and public health.^16^ The conceptual shift toward ecosystem thinking is underway; operational metrics have yet to follow.

Despite these challenges, evolving microbial control to reflect MoBE complexity offers clear benefits. Humans both affect and are affected by the microbiomes of the spaces they inhabit.^17^ Evidence linking environmental microbial exposure to immune development and allergic disease underscores that microbial exposure is multidimensional^18–20^ and host-specific, and our understanding of these relationships is rapidly improving. While infrastructure management cannot tailor microbial states to individual occupants, exposure consequences are understood to be broader than acute infection risk alone. Particularly for sensitive populations, ecological shifts in microbial communities may influence health through pathways not captured by pathogen-specific standards. Emerging insights into these interactions are poised to reshape microbial control strategies.

## Research Needs

The practical implication of embracing MoBE complexity in engineering design and operation of buildings is phased expansion, not regulatory upheaval. Pathogen suppression is the non-negotiable baseline, complemented by research that links ecological stability, resistance dynamics, and disturbance responses under real conditions to pathogen persistence, exposure pathways, and measurable health risk. Longitudinal data sets are needed to define normal variability, alongside mechanistic models that link operations to ecological outcomes and exposure science that translates microbiome shifts into health-relevant pathways, including beneficial effects. Only with these elements in place can microbiome-informed metrics be responsibly integrated into engineering decisions.

Federal investments recognize that infrastructure decisions shape microbial ecology and exposure pathways.^16^ The field is transitioning from descriptive characterization to intervention-aware systems thinking. The critical question is not whether microbiome-informed engineering will emerge, but whether it will do so guided by quantitative evidence and risk-based principles rather than reactive adaptation.

Pathogen-centric control transformed environmental engineering in the 20th century. In the 21st century, sustaining public health in increasingly complex infrastructure systems requires acknowledging that engineered environments function as microbial ecosystems. A health risk-grounded framework that incorporates ecological stability, resistance dynamics, and exposure considerations alongside pathogen suppression represents the logical next phase of public health engineering, explicitly linking ecosystem behavior and health outcomes. Building this foundation now ensures future integration is rigorous and protective of both acute and long-term health.
